# Trust and Language as Predictors of Service Use

**DOI:** 10.1093/geroni/igaa057.1848

**Published:** 2020-12-16

**Authors:** Lauren Ring, Allen Glicksman, Michael Liebman, Misha Rodriguez

**Affiliations:** 1 Philadelphia Corporation for Aging, Philadelphia, Pennsylvania, United States; 2 IPQ Analytics, LLC, Kennett Square, Pennsylvania, United States; 3 Asociación Puertorriqueños en Marcha, Philadelphia, Pennsylvania, United States

## Abstract

Research on older migrants often starts with a set of assumptions- including the importance of language as a barrier to care. A comparative approach allows us to examine these assumptions as they impact access to services for older migrants. Our study compared two groups of older migrants – Mandarin speaking Chinese and Spanish speakers from Puerto Rico. Through a series of focus groups we learned that although language can be a barrier to service access, the more important element in reducing disparities for older migrants is the level of trust between older adult and provider. For the older Chinese participants, the presence of a native speaker whom they trust is contrasted with a lack of trusted native Spanish speakers available to Puerto Rican elders, who must often rely on translators from various providers. We will use this example to help explain the differences in service use by these two communities. Part of a symposium sponsored by the International Aging and Migration Interest Group.

